# FLEX-DBS: a flexible wireless platform for bilateral constant-current deep brain stimulation in freely moving rodents

**DOI:** 10.64898/2026.10.09.757775

**Published:** 2026-10-12

**Authors:** Robert D. Graham, Meaghan C. Creed, Matt Gaidica

## Abstract

**Background:**

Preclinical deep brain stimulation (DBS) in rodents is central to understanding how DBS works, but most rodent DBS is delivered through a tether, which constrains behavior, home-cage experiments and uninterrupted stimulation. Most long-runtime wireless mouse stimulators achieve their runtimes by setting stimulation parameters before or around implantation and operating from supplies of about 3 V.

**New method:**

FLEX-DBS (Flexible Deep Brain Stimulation) is a head-mounted, battery-powered stimulator that delivers bilateral constant-current stimulation from improved Howland current pumps on ±5 V rails. Amplitude, pulse width and frequency are programmable in continuous and burst modes from an iOS application over Bluetooth Low Energy, and stimulation continues autonomously after the phone disconnects. The device measures 19.5 × 22.5 × 8.72 mm, weighs 3.71 g in operation and runs on two size-13 zinc-air cells, which are exchanged by briefly detaching the device, pausing stimulation only for the exchange.

**Results:**

Into a 1 kΩ bench load, delivered current increased approximately linearly with the amplitude setting and was repeatable from pulse to pulse, with an asymmetric biphasic waveform whose recovery phase ran at about one fifth of the stimulating amplitude. Battery current was 1.47–1.89 mA in standby and 3.15–3.42 mA during stimulation at amplitude settings of 60–400 µA, giving a projected three days of continuous stimulation per pair of cells. Firmware v0.5 left a net charge of 26–35 % of the stimulating-phase charge per pulse into 1 kΩ. Into 47 kΩ, within the in vivo load range, stimulating current was compliance-limited at 93 µA. Pulse-derived estimates of the effective load presented by five implanted platinum electrodes in three mice had weekly means of 43–54 kΩ over eight weeks (32–80 kΩ across individual sessions), placing the maximum regulated current near 90 µA (81–101 µA at the weekly means). Analysis of the current-pump network bounds load-dependent current error at 3–8 % for these loads. In vivo, we delivered cingulate FLEX-DBS at high amplitude settings intended to evoke a robust stimulation-associated behavioral phenotype in six mice. Time spent immobile in an open field rose from 46.5 % at baseline to 75.7 % during stimulation (a 62.7 % increase in the group mean) and remained elevated after stimulation ended; without a sham control, this increase cannot be separated from within-session habituation.

**Comparison with existing methods:**

Among the published wireless rodent stimulators compared here, several offer some of these capabilities, but none combines bilateral constant-current output, real-time wireless control that continues as autonomous stimulation after disconnect, and both continuous and burst modes; FLEX-DBS does so at the cost of runtime measured in days rather than weeks.

**Conclusions:**

FLEX-DBS is designed for untethered DBS experiments in which parameters must be set, changed and verified at the cage. Its measured power consumption permits a projected three days of continuous operation per cell pair; firmware v0.5 requires improved charge balancing before prolonged in vivo stimulation. The electrode load, rather than the programmable range, sets the usable stimulation amplitude in vivo. In vivo, stimulation at high amplitude settings was associated with increased immobility, consistent with effective stimulation through implanted electrodes; electrode impedance and delivered current were not measured in these mice.

